# Supplementary material for: Tracking the dynamics and allocating tests for COVID-19 in real-time: An acceleration index with an application to French age groups and départements
Source: PLoS One. 2021 Jun 1;16(6):e0252443. doi: 10.1371/journal.pone.0252443 (PMC8168842; doi:10.1371/journal.pone.0252443)
Supplement: S1 Appendix — (PDF) [file pone.0252443.s001.pdf]

S1 Appendix

A.1 Decomposing the Detection Effect of Tests

A country is divided into N regions, each indexed by $i = 1, \ldots, N$. For each region, data is available about the number of tested and positive persons, up to end date T. Denote $\{p_1^i, \ldots, p_T^i\}$ the historical times series of the new (per period) number of positive persons from date $t = 1$ to end date $t = T$. Similarly, $\{d_1^i, \ldots, d_T^i\}$ is the historical times series of new (per period) diagnosed/tested persons.

Denote $P_t^i = \sum_{\tau=1}^{t} p_\tau^i$ and $D_t^i = \sum_{\tau=1}^{t} d_\tau^i$ the cumulative numbers of positive and diagnosed persons up to date t. Finally, define $\tilde{P}_t^i = P_t^i / P_T^i$ and $\tilde{D}_t^i = D_t^i / D_T^i$ for $t = 1, \ldots, T$, which are the fractions of, respectively, positive and diagnosed persons at date t relative to that at end date. In more technical term, dividing the historical times series by the most recent entry amounts in our setup with non-negative numbers to perform min-max normalization (see See Han, Kalber, and Pei [4], section 3.5.2). Our object of interest is the relationship between $\tilde{P}^i$ and $\tilde{D}^i$ over time in the context of a pandemic, when testing is the only way to detect confirmed cases, which is depicted in the scatter-plot of Figure 2.

Suppose that the data $\{p_1^i, \ldots, p_T^i\}$ and $\{d_1^i, \ldots, d_T^i\}$ are used to estimate for each region a function f^i such that $\tilde{P}_t^i = f^i(\tilde{D}_t^i)$ for $t = 1, \ldots, T$. Because by definition $\tilde{P}_T^i = \tilde{D}_T^i = 1$ one has $f^i(1) = 1$. A first-order Taylor expansion at $\tilde{D}_T^i = 1$ gives:

$$\tilde{P}_t^i = 1 + (f^i)'(1)[\tilde{D}_t^i - 1] \tag{3}$$

Equation (3) shows that the derivative $(f^i)'(1)$ is essentially an elasticity, given the normalization of cumulative numbers by end date values: it measures how many positives follow an increase in the number of tests at end date, as a fraction of end date values. For example, values such that $(f^i)'(1) > 1$ mean that the pandemic is accelerating, since a given fraction of the total of tests performed up to date T is associated with a *larger* fraction of positives who are detected with those tests, in percentage of the cumulative number of positives at T. On the contrary, $(f^i)'(1) < 1$ implies that the pandemic is decelerating. This elasticity is what we have labeled the acceleration index and labeled ε_T in Section 2.1.

All of the above implies that, given the estimate of the first-order derivative, $\varepsilon_T \equiv (f^i)'(1)$, equation (3) can be rewritten in terms of the numbers of positive and tested persons, that is:

$$P_t^i - P_T^i = \frac{P_T^i}{D_T^i} \times (f^i)'(1) \times [D_t^i - D_T^i] \tag{4}$$

In other words, equation (4) can be used to decompose the effect of tests on positives *in levels*, that is, how many additional positives are detected given additional tests, between T and $T + dt$:

$$\underbrace{P_{T+dt}^i - P_T^i}_{\text{additional positives}} = \underbrace{(P_T^i / D_T^i)}_{\text{average positive rate}} \times \underbrace{(f^i)'(1)}_{\text{acceleration index}} \times \underbrace{[D_{T+dt}^i - D_T^i]}_{\text{additional diagnostics}} \tag{5}$$

Equation 5 is identical to equation 1 in Section 2.1, where ε_T is estimated as the ratio of variations of cases and tests between T and $T-1$. Similarly, the above decomposition in levels holds for any date $t < T$, as follows:

$$P^i_{t+dt} - P^i_t = \frac{P^i_T}{D^i_T} \times (f^i)' \left(\frac{D^i_t}{D^i_T}\right) \times [D^i_{t+dt} - D^i_t] \tag{6}$$

From equation (6), the effect of tests on positives in percentage terms from the perspective of date t is therefore written as:

$$\frac{P^i_{t+dt} - P^i_t}{P^i_t} = \frac{P^i_T/D^i_T}{P^i_t/D^i_t} \times (f^i)' \left(\frac{D^i_t}{D^i_T}\right) \times \left[\frac{D^i_{t+dt} - D^i_t}{D^i_t}\right] \tag{7}$$

The elasticity of the number of positives with respect to the number of tests is now, because it is evaluated at date t as opposed to end date T, the product of the derivative at the relevant point, times the ratio of average positive rates – that of date T over that of date t.

A.2 Decomposition and Acceleration index: Exponential Case

This section explores what the decomposition stated in Section A.1 reveals when time is assumed to be continuous and when the number of cases grows exponentially over time, as usually assumed in epidemiological models, of SIR type and related for example. Although typically absent in the latter strand of literature, we have to introduce tests and we assume that they also grow exponentially. More formally, using the notation in the previous section, suppose that the number of cases per unit of time is denoted by $p(t) = \alpha e^{\beta t}$ while the number of tests per unit of time is $d(t) = \gamma e^{\nu t}$, where the growth rates β and ν are assumed to be positive for the sake of illustration. Cumulated cases and tests are then noted $P(t) = \int_0^t p(\tau)\, d\tau$ and $D(t) = \int_0^t d(\tau)\, d\tau$, respectively. It is easy to derive, by straight integration, the expressions:

$$P(t) = \frac{\alpha}{\beta}\left(e^{\beta t} - 1\right), \; D(t) = \frac{\gamma}{\nu}\left(e^{\nu t} - 1\right) \tag{8}$$

It follows that our acceleration index is given, as function of time, by:

$$\varepsilon(t) = \frac{p(t)/d(t)}{P(t)/D(t)} = \frac{\beta}{\nu}\left(\frac{1 - e^{-\nu t}}{1 - e^{-\beta t}}\right) \tag{9}$$

From equation (9), then, one infers that two cases occur. When $\beta = \nu$, that is, when both cases and tests grow at the exact same rate, then our acceleration index equals 1 at all dates. When the two growth rates differ, however, $\varepsilon(t)$ converges, when t goes to infinity, to the ratio of growth rates β/ν, independently of the scale parameters α and γ. As an illustrative example, suppose that $\beta > \nu$, so that positives grow faster than tests. Then the pattern of our acceleration index $\varepsilon(t)$ over time will have two regimes: it first grows almost linearly and eventually reaches the upper bound $\beta/\nu > 1$. Obviously, in that case both the daily positivity rate $p(t)/d(t)$ and the average positivity $P(t)/D(t)$ grow over time, and the former quantity exceeds the latter all the time so that acceleration prevails. This closely resembles the pattern following early August to early October in Figure 3, as underlined in the main text.

A.3 Statistics for Age Groups

In Table 1 we report a few statistics for all age groups, as of October 25, 2020. In the second and third columns we report the numbers of cumulated cases and tests, respectively. The fourth column depicts that average positivity rate, defined as the ratio of cumulated cases and cumulated tests, while the actual test shares appear in the fifth column. Finally, the last column shows the share of cases by age group, which is defined as the ratio of cumulated cases.

Table 1: Some statistics for all age groups, as of October 25, 2020.

Age	Cum. cases	Cum. tests	Av. pos. rate	Test share	Case share
Less than 9 years old	765847	28549	3.73	5.11	2.72
Between 10 and 19 years old	1828655	128582	7.03	12.21	12.23
Between 20 and 29 years old	2598434	241206	9.28	17.35	22.95
Between 30 and 39 years old	2359316	173372	7.35	15.76	16.49
Between 40 and 49 years old	2078304	152898	7.36	13.88	14.55
Between 50 and 59 years old	1905289	136702	7.17	12.72	13.00
Between 60 and 69 years old	1461222	86848	5.94	9.76	8.26
Between 70 and 79 years old	1019982	50383	4.94	6.81	4.79
Between 80 and 89 years old	658065	34275	5.21	4.40	3.26
90 years old and more	297835	18368	6.17	1.99	1.75

A.4 Test Allocation across Départements: Actual vs Acceleration-based

Table 2: Allocation of tests in French départements - population vs acceleration, as of October 25, 2020. Data source: Agence Santé Publique France

French Dpt	Population shares	Observed tests shares	Acc. Weigths $\beta = 1$	Acc. Weigths $\beta = 3$
Ain	1.00	1.15	1.71	3.39
Aisne	0.83	0.65	0.91	0.50
Allier	0.52	0.31	1.00	0.68
Alpes-de-Haute-Provence	0.25	0.21	1.06	0.80
Hautes-Alpes	0.22	0.20	1.61	2.80
Alpes-Maritimes	1.68	1.36	0.78	0.31
Ardèche	0.50	0.50	1.48	2.18
Ardennes	0.42	0.60	1.03	0.74
Ariège	0.24	0.20	0.54	0.11
Aube	0.48	0.52	0.68	0.21
Aude	0.57	0.47	0.82	0.37
Aveyron	0.43	0.49	1.07	0.83
Bouches-du-Rhône	3.13	3.47	1.32	1.56
Calvados	1.07	1.06	0.89	0.48
Cantal	0.22	0.24	0.77	0.30
Charente	0.55	0.37	0.50	0.08
Charente-Maritime	1.00	0.73	0.51	0.09
Cher	0.47	0.34	1.37	1.74
Corrèze	0.37	0.27	0.72	0.25
Corse-du-Sud	0.24	0.23	0.71	0.24
Haute-Corse	0.27	0.29	0.69	0.22
Côte-d’Or	0.83	0.90	1.21	1.20
Côtes-d’Armor	0.93	0.63	0.52	0.09
Creuse	0.18	0.24	0.66	0.19
Dordogne	0.64	0.54	0.65	0.19
Doubs	0.83	0.89	1.07	0.83
DrÃŽme	0.79	0.81	1.52	2.38
Eure	0.93	0.56	1.00	0.67
Eure-et-Loir	0.67	0.53	1.09	0.87
FinistÃšre	1.41	0.94	0.64	0.18
Gard	1.15	1.22	1.50	2.27
Haute-Garonne	2.11	2.42	1.07	0.83
Gers	0.30	0.19	0.83	0.38
Gironde	2.45	1.59	0.87	0.45
Hérault	1.77	1.94	1.21	1.21
Ille-et-Vilaine	1.64	1.37	0.94	0.56
Indre	0.34	0.23	0.94	0.56
Indre-et-Loire	0.94	0.97	0.91	0.51
Isère	1.95	1.92	1.73	3.51
Jura	0.40	0.53	1.37	1.73
Landes	0.63	0.39	0.83	0.39
Loir-et-Cher	0.51	0.34	0.83	0.38
Loire	1.18	1.96	1.86	4.35

Table 3: Allocation of tests in French départements - population vs acceleration, as of October 25, 2020 (Cted). Data source: Agence Santé Publique France

French Dpt	Population shares	Observed tests shares	Acc. Weigths $\beta = 1$	Acc. Weigths $\beta = 3$
Haute-Loire	0.35	0.50	2.04	5.71
Loire-Atlantique	2.16	2.13	0.86	0.42
Loiret	1.05	1.36	1.04	0.75
Lot	0.27	0.18	0.58	0.13
Lot-et-Garonne	0.51	0.33	0.70	0.23
Lozère	0.12	0.14	1.84	4.23
Maine-et-Loire	1.26	1.18	0.99	0.66
Manche	0.77	0.54	0.63	0.17
Marne	0.88	0.75	0.99	0.66
Haute-Marne	0.27	0.22	1.26	1.34
Mayenne	0.48	0.27	0.80	0.35
Meurthe-et-Moselle	1.13	1.27	0.89	0.47
Meuse	0.29	0.24	0.70	0.24
Morbihan	1.16	0.80	0.71	0.24
Moselle	1.61	1.84	0.96	0.61
Nièvre	0.32	0.22	0.81	0.36
Nord	4.03	7.70	1.29	1.44
Oise	1.28	0.95	0.96	0.60
Orne	0.44	0.24	1.16	1.04
Pas-de-Calais	2.27	2.57	1.14	1.00
Puy-de-Dôme	1.01	1.20	1.25	1.32
Pyrénées-Atlantiques	1.05	1.29	0.87	0.45
Hautes-Pyrénées	0.35	0.30	1.13	0.98
Pyrénées-Orientales	0.73	0.77	0.98	0.63
Bas-Rhin	1.74	2.92	0.81	0.35
Haut-Rhin	1.18	1.17	0.58	0.13
Circonscription dép. du Rhône	2.85	4.12	1.60	2.79
Haute-Saône	0.37	0.32	0.89	0.47
Saône-et-Loire	0.86	0.77	1.55	2.53
Sarthe	0.88	0.65	0.87	0.45
Savoie	0.67	0.75	2.00	5.39
Haute-Savoie	1.25	1.50	1.78	3.82
Paris	3.38	4.15	1.06	0.80
Seine-Maritime	1.94	1.86	1.09	0.87
Seine-et-Marne	2.17	1.09	1.04	0.75
Yvelines	2.23	1.71	1.22	1.22
Deux-Sèvres	0.58	0.50	0.77	0.30
Somme	0.89	0.62	0.76	0.29
Tarn	0.60	0.46	1.24	1.30
Tarn-et-Garonne	0.40	0.36	1.22	1.21
Var	1.64	1.78	0.94	0.56
Vaucluse	0.87	0.93	1.39	1.82
Vendée	1.04	0.82	0.75	0.28

Table 4: Allocation of tests in French départements - population vs acceleration, as of October 25, 2020 (Cted). Data source: Agence Santé Publique France

French Dpt	Population shares	Observed tests shares	Acc. Weigths $\beta = 1$	Acc. Weigths $\beta = 3$
Vienne	0.68	0.54	0.88	0.46
Haute-Vienne	0.58	0.61	1.00	0.68
Vosges	0.57	0.59	0.77	0.31
Yonne	0.52	0.26	0.87	0.44
Territoire de Belfort	0.22	0.15	0.72	0.25
Essonne	2.01	1.34	1.17	1.07
Hauts-de-Seine	2.49	2.71	1.13	0.97
Seine-Saint-Denis	2.51	1.97	1.36	1.69
Val-de-Marne	2.15	1.90	1.04	0.77
Val-d'Oise	1.90	1.68	1.52	2.37
